# Patterns of MRI Findings in Patients with Chronic Headache: A Retrospective Study from a Private Diagnostic Center in Addis Ababa, Ethiopia

**DOI:** 10.4314/ejhs.v32i1.2S

**Published:** 2022-10

**Authors:** Abebe Mekonnen W/Yohannes, Tequam Debebe W/Hawariyat, Tesfaye Kebede Legesse

**Affiliations:** 1 Department of Radiology, College of Health Science, Addis Ababa University

**Keywords:** Chronic headache, Neuroimaging, MRI, CT-scan, Ethiopia, Africa

## Abstract

**Background:**

Headache is one of the most common complaints that lead the patient to seek medical advice however only a few patients with recurrent headaches have a secondary cause like intracranial mass. The appropriate utilization of neuroimaging is important to rule-out secondary cause of headache in resource-limited regions. The objective of this study is to describe the patterns of MRI findings in the evaluation of patients with chronic headache and to determine the clinical variables helpful in identifying patients with intracranial lesions.

**Materials and Methods:**

This cross sectional study was conducted among 590 selected patients who underwent an MRI scan of the head from September 2016 to January 2018 at Wudassie Diagnostic center in Addis Ababa, Ethiopia. Siemens Magnetom 0.35T MRI was used.

**Results:**

Out of 590 patients, 372 (63.1%) were females and 218 (36.9%) were males; 300 (50.8%) patients with the mean age of 38.6 ± 0.5 years and a median of 37 ± 16.7years have normal brain MRI and 290(49.2%) have abnormal brain MRI reports. The abnormal findings further divided into non-significant findings were 166(28%) that did not alter patient management and clinically significant findings were 124 (21%) which included by decreasing order of frequency tumors, infection, hydrocephalus, hemorrhage, and vascular abnormalities.

**Conclusion:**

It was 1.3 times higher rate of positive brain MR findings in patients who had headaches plus abnormal neurologic findings as compared to patients without neurologic abnormality (P-value = 0.01). There is a high rate of significant abnormal MRI findings in this study as compared to studies from developed nations.

## Introduction

Headache is one of the most common complaints that lead the patient to seek medical advice or treatment from their physician however only about 10% of patients with recurrent headaches have secondary cause (1, 2). The primary headache disorders may include migraine, cluster and tension-type headaches account for the majority of headaches. The secondary headaches, which are those with underlying pathology (e.g., tumor, vascular malformation, or infection) are far less common (3). The majority of patients who present to the outpatient clinic have no serious underlying intracranial cause (4). Although patients with recurrent headaches without neurologic deficit have no intracranial pathology, many patients undergo an evaluation with computed tomography (CT) and magnetic resonance (MR) imaging to exclude important abnormalities. Previous studies have demonstrated that CT is of extremely low yield in patients who undergo imaging for a chronic headache without neurologic abnormality (5–7). MRI is more sensitive than CT for the evaluation of brain parenchymal lesion(8). Headache disorders are in the top ten-and possibly the top five causes of disability worldwide. Globally, the prevalence of the adult population with active headache disorders is 46% for headache in general, 11% for migraine, 42% for tension-type headache and 3% for chronic daily headache(9). Headache is also a common problem in children. The reported rates of headache prevalence during childhood range from 26.6% to 93.3%(10). In a study from Turkey, KarlıN et al. reported that the prevalence of recurrent headache in adolescents aged between 12 to 17 years was 52.2% (11).

Some clinical features make headaches in the tropics different from the rest of the world. Headaches due to organic causes, mainly infection, are common in the tropics. Contrary to beliefs in the past, the primary headache like a migraine is not a rare cause among Africans(12). Mengistu G etal in the study from Ethiopia also reported that the overall one-year prevalence of primary headache disorders was 21.6%. The following types of primary headache accounts migraine were 10%, migraine without and with aura were 6.5% and 2.6 % respectively. The other types which include probable migraine was 0.9%, tension-type of headache was 10.4%, the frequent episodic tension-type headache was 8.2% followed by the infrequent tension-type headache of 2.2% as well as cluster headache was 1.3%(13). In most countries in Africa there are often delays in seeking medical care and in the diagnosis of organic causes of headache. As a result, the headache is often unrecognized and underdiagnosed. Its frequency is therefore not yet accurately determined (12).

American Academy of Neurology in 2000 had published practice guidelines for imaging in headache(5,7,14,). Neuroimaging recommendations for non-acute headache includes patients with abnormal neurologic examinations and patients with atypical headache features for primary headache or high risk patients like immune deficiency, cancer and high risk population for intracranial disease. Neuroimaging is not usually recommended in patients with migraine or tension type headache with normal neurologic examination.

Considering the burden of headache and its economic impact, it is crucial to understand the imaging findings of patients having chronic headache to understand the causes and in what additional clinical findings that the clinician expects positive imaging findings. Therefore, this research was done to analyze the patterns of MRI findings in patients who have chronic headache. It also analyzed the relationship between clinical history mainly additional neurological abnormality and imaging findings.

## Materials and Methods

**Study design**: This is a retrospective study done by reviewing imaging records of patients who had an MRI scan for a complaint of chronic headache from those who visited the center during the period September 2016 to January 2018. Data was collected from Wudassie Diagnostic Center, Addis Ababa, Ethiopia. Wudassie Diagnostic Center is one of the private diagnostic imaging centers in Addis Ababa City. Patients usually referred for imaging service MRI and CT-scan from different health institutes of the capital city and other regions of the country.

In this cross sectional study a representative sample of 590 eligible study subjects were included in our review.

All patients for whom MRI was done for headache which stayed for more than a month were included in the study. Patients who have a history of trauma and head surgery were excluded from the study.

A structured questionnaire was prepared which contain demographic information, duration of headache and clinical findings like neurological symptoms and neurologic deficits. Data were then collected by a senior registered nurse who was given a short-term training on data collection. The imaging center archives are well organized with date of the examination which include, day, month and time of examination. The archives contain patient request form from the refering physician which contains relevant clinical information and type of requested examination. There is also a format which the center is using to collecte all the relevant clinical information, duration of illness, previous surgical intervention, MRI compatibility and recent use of drugs etc. All indivisual patient documents; physician request, completely filled form prepared by the center and copy of the radiologist report are kept both with hard and soft copy so that accessing patient record was not difficult.

All patient identifiers were not used during data collection instead codes were given on both the copy of the report and the questionnaire to trace the report in case the questionnaire was incompletly filled. The filled questionnaire were checked for completness and those who are incomplete were traced using the given code and completed.

The authors performed a retrospective review of MRI reports which were dictated by two licensed radiologists. The MR images were interpreted by two of the authors who have more than 10 years of experience.

Ethical clearance was obtained from department of radiology; College of Health Sciences; Addis Ababa university ethical committee. Permission to use the data was given from the diagnostic center where the data was collected.

Statistical Analysis

Statistical analysis was done using Statistical Package of Social Science (SPSS Version 21). Data comparison was done by applying specific statistical tests i.e. Chi-Square test to find out the statistical significance of the comparisons. Qualitative variables were compared using proportions. Significance level was fixed at p < 0.05.

The reports of all head MRI of patients of chronic or recurrent headache reviewed and the MR imaging results in to normal and abnormal. The MR abnormal findings further divided in to a) those with minor abnormality or insignificant abnormalities such as non-specific white matter changes like chronic ischemia, small arachnoid cyst, prominent perivascular CSF spaces which neither explained the reason for headache nor changed the clinical or therapeutic approach and b) Those with clinically important intracranial abnormality or significant abnormality such as neoplastic lesion, hematoma, hydrocephalus, infection, vascular abnormalities (Dural venous thrombosis, arteriovenous malformation ) which may result in chronic or recurrent headache or change the clinical or therapeutic approach or require further action.

Subjects were also further divided in to two groups I and II based on additional neurologic symptoms or sign as patients with no neurologic abnormalities and patients with additional neurologic symptoms or deficit respectively. MRI findings were evaluated for any intra and extra cranial pathology.

Equipment: MR imaging.

All subjects underwent MR scanning by the same Siemens Magnetom C 0.35T machine. The images include Axial TSE T2 (slice thickness/slice gap of 5/1.5mm, TR/TE of 6210msec/114msec, Echo train length (ETL) 11, Matrix 256x163), Axial SE T1 (slice thickness/slice gap of 5/1.5mm, TR/TE of 540msec/11msec, ETL-1, Matrix 256x138), Sag SE T1 (slice thickness/ slice gap of 5/1.5mm, TR/TE of 428msec/11msec, ETL-1, Matrix 256x166), Cor FLAIR (slice thickness/ slice gap of 5/1.5mm, TR/TE of 8574msec/79msec, ETL-7, Matrix 256x159), Axial epi DWI with ADC-map (slice thickness/ slice gap of 8/2mm, TR/TE of 6436msec/180msec, ETL-1, Matrix 96x96).

The MRI was done with and without contrast depending on the clinical indication and findings on pre-contrast study. If contrast examination was done, images were taken in Axial, Sagittal and Coronal SE T1 planes and gadodiamide (GdDTPA-BMA) equiv.0.5mmol (Omniscan; of GE Healthcare) was used with dosage of 0.1mmol/kg of bodyweight (equivalent to 0.2ml/kg BW).

## Results

A total of 646 patients who underwent MRI scan of head from September 2016 to January 2018. Out of 646 cases, 56 were excluded because they did not fulfill the inclusion criteria. The rest 590 cases were analyzed. Among the 590 patients, 218 patients underwent intravenous contrast administration as based on clinician's request and/or when radiologist required it for better imaging and characterization of the pathology.

**Figure 1:**
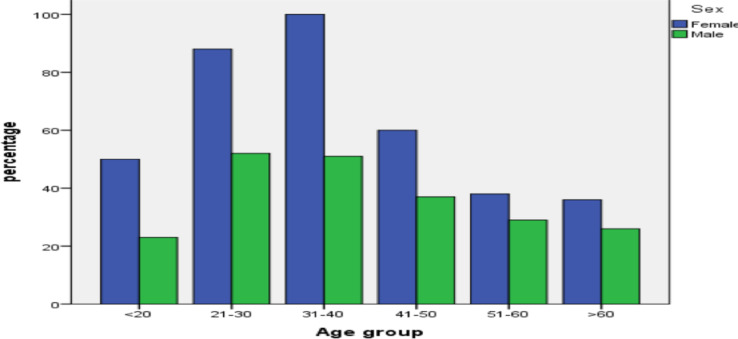
Sex and age distribution of cases

Out of 590 patients 372 (63.1%) were females and 218 (36.9%) were males; 300 (50.8%) patients have normal brain MRI and 290(49.2%) have abnormal brain MRI reports. The age group ranged from 3 years to 88 years with the mean of 38.6 ± 0.5 years and median of 37 ± 16.7 years (Figure 1).

Close to 10% of cases (n= 58) were hypertensive, 4.9% of cases (n=29) were diabetic and 3.7% of cases (n= 22) were HIV positive. Fever was recorded in only 1 % of cases (n= 6). Additional neurologic abnormalities other than headache was recorded in 31.4 % of cases (n=185); Out of these monoplegia/paraplegia in 2.2% (n=13), vertigo/ataxia in 12.2% (n=72), Seizure in 5.8% (n=34), hemiplegia in 8% (n=47), numbness/tingling sensation in 2% (n=12), decreased vision in 10.2% (n=60) and neck stiffness in 0.5% of cases (n=3). The rest of patients, 405 (68.6%) with chronic headache have no neurological abnormalities. Patients with headache who have additional neurological abnormality (n=185) are more likely to have abnormal imaging findings than those who have no neurological abnormality (P-value = 0.01). More than half or 300 patients (50.8%) of imaging findings were normal and 166(28%) were having no clinically significant imaging abnormalities. Among those who have no clinically significant abnormal imaging findings, more than half have non-specific white matter changes which is highest rate from this group, n= 78 (26.9%), and others include old infarction or encephalomalacia, Sino-nasal findings like scattered mucosal thickenings or retention cysts, papilledema or optic nerve atrophy, other miscellaneous findings like small arachnoid cyst, demyelinating disease, enlarged perivascular CSF spaces which neither explained the reason for headache nor changed the clinical or therapeutic approach (Table 1).

**Table 1:** Distribution of radiologic findings according to presence or absence of additional neurologic findings or deficit from the history

CASES			Imaging findings		Total
			Normal	Abnormal	
Neurologic deficit	Absent	Number	221	184	405
		% with no additional neurologic abnormality.	54.6%	45.4%	100.0%
	Present	Number	79	106	185
		% with additional Neurologic abnormality	42.7%	**57.3%**	100.0%
Total		Number	300	290	590
			50.8%	49.2%	100.0%

P-value = 0.010

**Figure 2:**
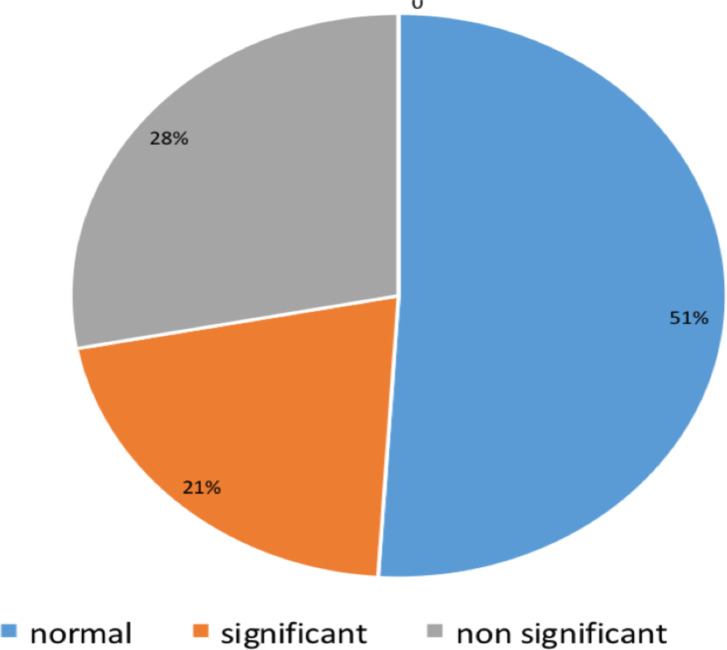
Distribution of cases according to significance radiologic diagnosis to patient chief complaint.

The other group are cases with significant findings constitute 124 (42.8%) of the abnormal findings which may result in chronic or recurrent headache or change the clinical or therapeutic approach or require further action (Figure 2). These are neoplastic lesion (intra- and extra axial tumors, pituitary macroadenoma), intracranial hemorrhage (subdural or brain parenchymal hemorrhage), hydrocephalus, infection (tuberculoma, toxoplasmosis, meningitis), vascular abnormalities (Dural venous thrombosis, arteriovenous malformation, aneurysm).

The radiological diagnosis of intracranial tumors was made for 75 (12.7%) of the patients. From these extra-axial tumors constitute 41 (6.9%) which include meningioma, Schwannoma and others and 20 (3.4%) of patients have intra-axial tumors like high-grade glioma and metastasis. Others like pituitary macroadenoma and skull vault or skull base tumors account for 11 (1.9%) and 3 (0.5%) of patients respectively.

**Figure 3:**
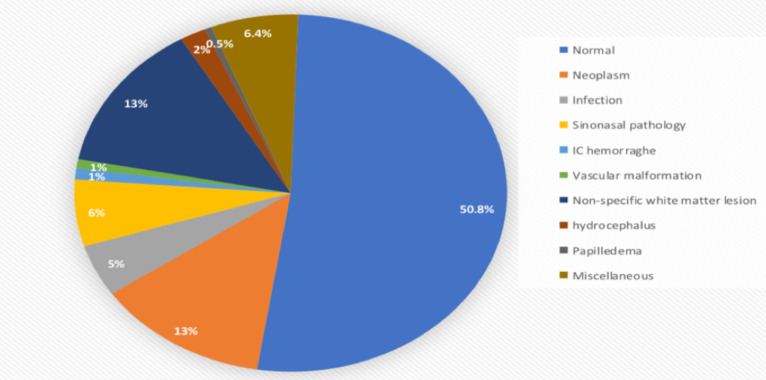
Distribution of cases according to radiologic diagnosis

The number of cases with radiological diagnosis of intracranial infection like tuberculoma, toxoplasmosis and others account for 27 (4.6%) patients. Among the 27 cases who had infection 19 (3.2%) had tuberculoma. The other radiologic diagnosis among clinically significant cases are hydrocephalus, intracranial vascular abnormalities and intracranial hemorrhage are 11 (1.9%), 5 (0.8%) and 6(1%) respectively (Figure 3).

## Discussion

We reviewed imaging records of 646 patients from which we excluded 56 reports because they do not fulfill inclusion criteria. 590 patients were included in the study comprising of 372 (63.1%) females and 218 (36.9%) males with a mean age of 38.6. Our study revealed that more than two-third (79%) of brain MRI studies done for patients with chronic headache are either normal or showed non-significant imaging findings which neither explained the reason for headache nor changed the clinical or therapeutic approach to patients. Those patients with chronic headache who had associated neurological abnormalities are more likely to have abnormal brain MRI findings than those who have no neurological deficit.

Headache is a common clinical condition, which is associated with different pathologic condition. Globally, the prevalence of the adult population with active headache disorders are 46% for headache in general, 11% for migraine, 42% for tension-type headache and 3% for chronic daily headache. Even community-based survey in Addis Ababa, Ethiopia revealed the one-year prevalence of headache to be 21.6% with tension headache and migraine headache being the most common accounting 10.4% & 10 % respectively. Even if headache is a common problem, most imaging workups with CT & MRI findings are either normal or non-significant lesions which neither explain the headache nor change the subsequent treatment plan(16–18). This was also shown in our current study which revealed more than two-third to have either normal or not clinically -significant imaging findings. Some of the imaging findings can also be found in MRI of asymptomatic patients (19).

Our study also showed those cases who have chronic headache and associated neurological deficits are more likely to have abnormal MRI findings than those without neurological deficit. This was also demonstrated in other studies (20). In this regard then imaging may not be important in patients with chronic headache without neurological abnormality. So careful clinical history and physical examination should be used to pick those cases who need further imaging evaluation. Use of clinical warning criteria (CWC) which includes Increase in the intensity and frequency of headache, abrupt onset of headache, persistence of headache despite analgesics, alteration of the characteristics of headache and presence of focal neurological symptoms useful in identifying Patients with secondary headaches and predicting intracranial pathology using CT (20). The rate of detection of positive finding was quite higher among patients who meet clinical warning criteria CWC criterion (17, 20). This will reduce the burden to the radiology department in most developing countries like Ethiopia where imaging modalities are unavailable and expensive if they are available. This will also avoid unnecessary cost and anxiety to patients that may arise from identifications of non-significant imaging findings.

The significant imaging findings in our series is 21% with intracranial tumors accounting 12.7% of cases which is higher than other studies done in India and other developed nations (17). The discrepancy can be explained by the fact that Health seeking behavior of our community is inadequate(13) and access to healthcare is poor. Even if patients have access to healthcare, imaging modalities, especially CT/MRI are unavailable or are expensive for most patient. On the other hand, wide availability of imaging modalities and health seeking behavior of developed nations result in overuse of imaging.

Intracranial infections are also one of the findings in our series, which accounted for 4.6% of cases of which the majority were tuberculoma followed by toxoplasmosis. This is not surprising knowing that Ethiopia is one of the countries with infection including HIV being one of the challenging health care problem like other tropical countries (12).

Even if imaging is important in the workup of patients having chronic headache, proper clinical history and physical examinations including the clinical warning criteria (CWC) should be used to identify those cases who need imaging evaluation. Since imaging findings are more likely to be positive in patients with headache and associated neurological findings, patients should be examined for neurological abnormalities. In our country, infections should also be considered in the differential diagnosis of patients with chronic headache.

This study has number of limitations. It has retrospective nature and most of the patients coming from different health institutions of Addis Ababa city or nearby towns and the record of complete neurologic evaluation could not be obtained. The majority of the patients referred with unspecified headache and not classified according to the standard international classification of headache disorder by referring physicians which limits the scope of sub-group analysis and our ability to give a practical recommendation.

Our study has also number of strengths. This is the first study in Ethiopia which attempts to show the common causes of chronic headache based on MRI evaluation. We used large sample sizes of patients referred to the imaging center for MR imaging of headaches from different health institutes and standard statistical tools that will ensure the reliability of our estimates.

In conclusion the most common causes of chronic headache were brain tumors, intracranial infection, hydrocephalus, intracranial hemorrhage, and vascular abnormalities. The MRI study is more likely to be abnormal in those who have associated neurological signs and symptoms than in those who have no additional clinical neurological findings. The authors recommended proper clinical evaluation for the appropriate use of imaging in patients with chronic headache.

## Figures and Tables

**Table 2:** Distribution of radiological diagnosis according to gender

Radiological diagnosis	Sex		Total
		
	Female	Male	
Normal	200 (53.8%)	100 (45.9%)	300 (50.8%)
Neoplasms	45(12.1%)	30 (13.8%)	75(12.7%)
Infections	19(5.1%)	8 (3.7%)	27(4.6%)
sinonasal pathology	16(4.3%)	19 (8.7%)	35(5.9%)
IC-hemorrhage	3(0.8%)	3 (1.4%)	6(1%)
Vascular lesions	3(0.8%)	2 (0.9%)	5(0.8%)
Non-specific white matter lesions	44(11.8%)	34 (15.6%)	78(13.2%)
encephalomalacia	4(1.1%)	8 (3.7%)	12(2%)
Hydrocephalus	8(2.2%)	3 (1.4%)	11(1.9%)
Papilledema	1 (0.3%)	2 (0.9%)	3(0.5%)
Miscellaneous	29 (7.8%)	9 (4.1%)	38(6.4%)
Total	372 (100%)	218 (100%)	590(100%)
